# Conserved Cysteines of a Putative Zinc Finger Motif in P48 Are Important for the Nuclear Egress of Nucleocapsids and the Envelopment of Occlusion-Derived Virions

**DOI:** 10.3390/v17030434

**Published:** 2025-03-18

**Authors:** Xiaoyan Ma, Jiang Li, Manli Wang, Zhihong Hu, Huanyu Zhang

**Affiliations:** State Key Laboratory of Virologyand Center for Biosafety Mega-Science, Wuhan Institute of Virology, Chinese Academy of Sciences, Wuhan 430071, China; xiaoyanmacas@gmail.com (X.M.); lijiang@wh.iov.cn (J.L.); wangml@wh.iov.cn (M.W.)

**Keywords:** AcMNPV, P48, zinc finger motif, conserved cysteine, infectivity

## Abstract

The open reading frame 103 (*p48*) of Autographa californica multiple nucleopolyhedrovirus (AcMNPV) is one of the 38 core baculovirus genes. *p48* has been shown to be essential for the production of infectious budded virions (BVs), nuclear egress of nucleocapsids, envelopment of the nucleocapsid, and embedding of occlusion-derived virions (ODVs) into occlusion bodies (OBs). However, the structure–function relationship of P48 remains unclear. In this study, we showed that four conserved cysteines (C127, C130, C138, and C141) in P48 may form a zinc finger motif based on a predicted structure analysis, and we investigated the roles of these cysteines in P48 function. AcMNPV bacmids lacking *p48* or containing mutated *p48* were generated. Transfection/infection assays showed that C127, C130, C138, and C141 in P48 were crucial for infectious BV production. Electron microscopy analysis further confirmed that these four cysteines played critical roles in the transport of nucleocapsids out of the nucleus for BV production, and in ODV envelopment. These results demonstrate that the conserved cysteines C127, C130, C138, and C141, related to the putative zinc finger motif, are critical for P48 function in baculovirus infection.

## 1. Introduction

Baculoviruses belong to a class of large DNA viruses that infect insects and are one of the most commonly used vectors for exogenous protein expression. Compared to other viruses, they have a unique biphasic life cycle, producing two types of viral particles. One type is the budded virus (BV), responsible for systemic infection between cells within the insect host. The other type is the occlusion-derived virus (ODV), which is embedded in the viral occlusion bodies (OBs) within the infected cell’s nucleus and is responsible for horizontal transmission between insect hosts. A set of genes that are conserved across all baculoviruses, known as core genes, have been identified, which are essential for critical events in the baculovirus life cycle. In-depth analysis of these core gene functions will deepen our understanding of baculovirus biology and help optimize the efficiency and quality of baculovirus-based systems for expressing exogenous proteins.

*p48* is one of the 38 baculoviral core genes, conserved in all sequenced baculovirus genomes [1]. In Autographa californica multiple nucleopolyhedrovirus (AcMNPV), P48 is encoded by ORF103 (*ac103*) and plays a crucial role in the virus life cycle, particularly in the formation and propagation of BV and ODV morphogenesis [2]. The deletion of *p48* severely impairs the nuclear egress of nucleocapsids, a critical step in BV production [2]. This transport is thought to be facilitated by interactions between P48 and proteins in the ESCRT-III complex, which are involved in membrane scission and egress processes [3]. Additionally, P48 is necessary for the efficient formation of intranuclear microvesicles, which are critical for ODV production [4]. P48 interacts with another viral protein, Ac93, which is also required for the efficient formation of intranuclear microvesicles [5]. Ac93, in turn, associates with components of the ESCRT complex [6]. In addition, five conserved sites (N318, V319, C320, R321, and I323) in P48 were identified to be associated with interactions with Ac93, and when these sites were mutated together, the nuclear egress of nucleocapsids and the formation of intranuclear microvesicles were blocked [5]. Still, the structure–function relationship of P48 remains unclear.

In this study, we demonstrate that P48 contains a putative zinc finger motif, as predicted by AlphaFold3, which is composed of four conserved cysteines. To further investigate its role, we generated *p48* knockout (KO) bacmids and a series of point-mutant bacmids to assess their impact on the viral life cycle. Our results indicate that the conserved cysteines of the zinc finger motif are essential for P48’s function at multiple stages of baculovirus infection.

## 2. Materials and Methods

### 2.1. Bioinformatics Analysis and Structure Prediction of P48 Homologs

Amino acid sequence alignment of 168 available P48 homologs from baculoviruses was performed using MAFFT v7.453 software [7]. Phylogenetic analysis of P48 homologs was performed using IQ-TREE [8]. The best-fit model was determined using the ModelFinder algorithm within IQ-TREE 1.6.12, and 1000 ultrafast bootstrap replicates were used to assess the reliability of the tree. The three-dimensional (3D) model of P48 homologs was predicted de novo using the AlphaFold3 server [9]. The domain topology of AcMNPV P48 was generated using TopDraw -Version: Sep 2002 [10], and functional domains were predicted using the Dali [11] and Foldseek servers [12], respectively. To analyze the evolutionary conservation of P48, the alignment was submitted to the Consurf server [13,14] to obtain conservation scores for each residue. These scores were mapped onto the protein structure to identify conserved regions relevant to the function of P48.

### 2.2. Generation of p48KO and Recombinant Viruses

*p48*-knockout AcMNPV bacmid (*p48*KO) was generated using AcBac-syn (AcBac) as the parental bacmid [15] by replacing the nucleotides (nt) 268 to 977 of *p48* with a bleomycin resistance gene cassette amplified from the pIZ/V5-His plasmid (Invitrogen, Waltham, MA, USA) through homologous recombination in *Escherichia coli*. Positive clones were selected through resistance screening. Fragments containing the native *p48* promoter and *p48* ORF were amplified and cloned into the pFastBac Dual plasmid (Invitrogen) as the donor plasmid for constructing the *p48*-repaired bacmid (*p48*Rep). Point-mutant donor plasmids of the conserved cysteines were generated using overlapping polymerase chain reaction (PCR) with the *p48*-repair donor plasmid as the template. Repaired and site-mutated bacmids were generated via transposition at the *hr4a* locus [15] using the Bac-to-Bac system [16].

Transfection and infection assays were conducted to evaluate the effects of the recombinant bacmids on viral replication. Purified DNA (1 μg) was transfected into 2 × 10^6^ Sf9 cells using Cellfectin (Gibco, Waltham, MA, USA) according to the manufacturer’s protocol. For the infection assay, cell supernatants were collected 96 h post-transfection and used to infect 2 × 10^6^ Sf9 cells. The transfected/infected cells were imaged using an EVOS fluorescence microscope (Thermo Fisher Scientific, Waltham, MA, USA).

### 2.3. Viral Titer Measurement and Growth Curve Analysis

The viral titers of C141S, C229S, C320S, and *p48*Rep P0 generation viruses, collected from the cell culture supernatant at 96 h post-transfection, were determined by measuring the infectivity of the produced BV using the endpoint dilution method. For growth curve analysis, cells were infected at a multiplicity of infection (MOI) of 5, or with C141S at an MOI of 0.02. Samples were collected every 24 h post-infection (h p.i.) for up to 96 h to assess virus titer. Virus titers were determined by the endpoint dilution method and expressed as TCID_50_/mL. The assay was performed in triplicate. Statistical analysis of the titer at 96 h p.i. between each group and the *p48*Rep control was conducted by Student’s t-test.

### 2.4. Electron Microscopy

Sf9 cells were transfected with recombinant bacmids and harvested at 72 h post-transfection. The samples were prepared as previously described [17] and analyzed by electron microscopy (Talos L120C, Thermo scientific) with an accelerating voltage of 120 kV.

## 3. Results

### 3.1. Structure Prediction Suggests P48 Contains a Conserved Putative Zinc Finger Motif

**Figure 1:**
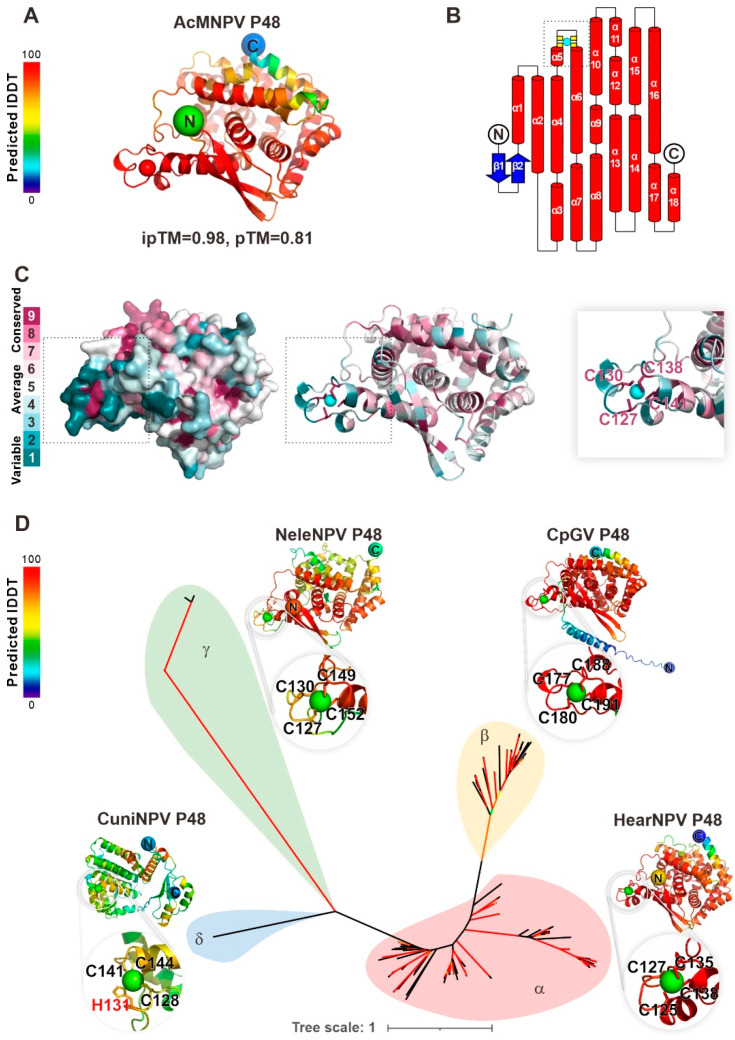
Baculovirus P48 homologs contain a conserved putative zinc finger motif. (**A**) AlphaFold3 predicted the structure of AcMNPV P48, with local accuracy assessed by IDDT scores (red: high, blue: low). (**B**) P48’s secondary structure includes β-strands (blue arrows) and α-helices (red cylinders). The zinc finger motif, with key residues C127, C130, C138, and C141 (yellow), likely binds a metal ion (cyan). (**C**) Consurf analysis shows high conservation of zinc-coordinating residues (dark purple), underscoring their functional importance. The zinc finger motif region is indicated by a gray dashed box. (**D**) An unrooted phylogenetic tree of baculovirus P48 homologs, supported by AlphaFold3 predictions, reveals evolutionary relationships. Representative structures from Helicoverpa armigera nucleopolyhedrovirus (HearNPV, alphabaculovirus), Cydia pomonella granulovirus (CpGV, betabaculovirus), Neodiprion lecontei nucleopolyhedrovirus (NeleNPV, gammabaculovirus), and Culex nigripalpus nucleopolyhedrovirus (CuniNPV, deltabaculovirus) highlight the conserved zinc finger motif. Branches with SH-aLRT > 80% or ultrafast bootstrap > 95% (red) indicate high confidence.

To analyze the molecular function of P48, its 3D structure of AcMNPV P48 was predicted using Alphafold3. The predicted template modeling (pTM) score and interface predicted template modeling (ipTM) score were 0.81 and 0.98, respectively, indicating the high confidence and high quality of the overall prediction (Figure 1A). As depicted in the predicted model, P48 contains two β-sheets in its N-terminus, followed by 18 α-helices (Figure 1B). The structure revealed a loop located between the N-terminal α5 and α6 helices, which contains a putative C4-type zinc finger motif with four cysteine residues: C127, C130, C138, and C141. The ConSurf server was used to assess the evolutionary conservation of all 168 available P48 homologs from different baculoviruses. The analysis revealed that the zinc finger motif is highly conserved across P48 homologs, and the residues C127, C130, C138, and C141, which coordinate the zinc ion, are also highly conserved, indicating their functional importance. In contrast, the loop harboring the zinc finger motif displayed lower conservation, indicating variability (Figure 1C). The zinc finger motif is structurally conserved in all the predicted structures of P48 homologs from alpha-, beta-, gamma-, and deltabaculoviruses (Figure 1D), although there is a slight difference in the deltabaculovirus, in which, instead of a C4 zinc finger motif, a C3H1 motif was identified. The conservation of the zinc finger motif suggests its importance for the function of the P48 protein during baculovirus infection.

### 3.2. Conserved Cysteines in Baculoviral P48 Homologs

**Figure 2:**
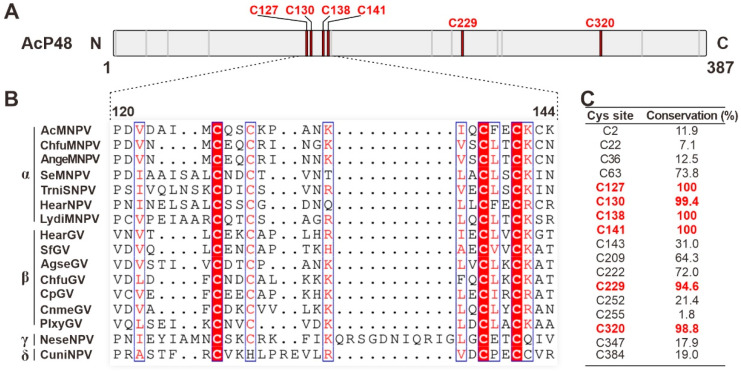
Conserved cysteines in P48 homologs. (**A**) Diagram of the primary sequence of AcMNPV P48. The locations of cysteines are indicated with short lines, with the highly conserved cysteines indicated as thick red lines. (**B**) Sequence alignment of P48 homologs from representative viruses from baculoviruses; only the region covering the zinc finger motif is presented. The sequences were aligned with MAFFT and edited with ESPript. Conserved cysteine sites are highlighted in red. (**C**) The conservation of each cysteine residue (shown in percentages from a total of 168 P48 homologs) in P48 is listed in the table. Sites with conservation greater than 90 are indicated in red.

AcMNPV P48 contains 17 cysteines. To further analyze the conservation of cysteines in P48, sequence alignment was performed with all 168 available P48 homologs from the sequenced baculoviruses (Figure 2B shows 16 representative sequences from four viral genera). The results showed that 6 of the 17 cysteines are highly conserved in all the available P48 homologs (Figure 2C). These include four zinc finger motif-related cysteines: C127, C130, C138, and C141. Additionally, C229 and C320 also show high conservation. The conservation of these cysteines in P48 suggests their importance for P48 function. Therefore, we aim to explore the role of these cysteines through mutagenesis analysis.

### 3.3. Zinc Finger Motif-Related Cysteines of P48 Are Critical for BV Production

**Figure 3:**
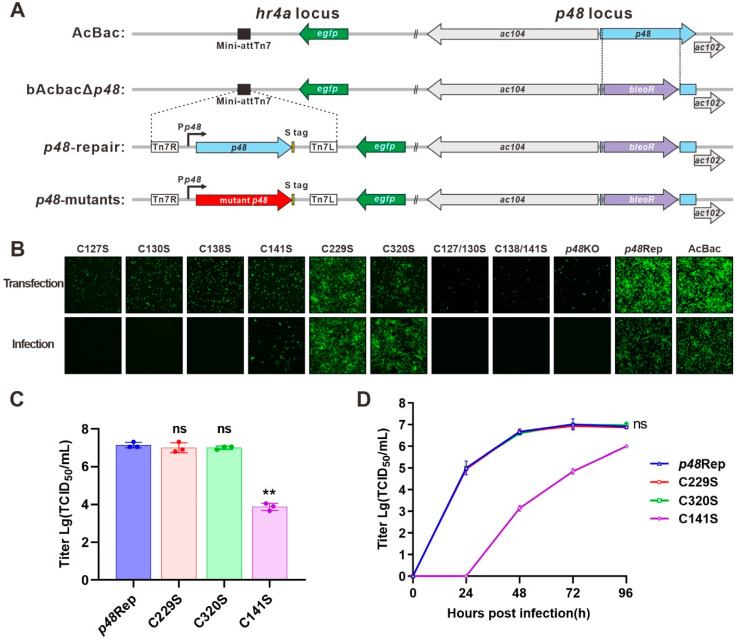
Construction and transfection–infection assay of recombinant bacmids. (**A**) Schematic diagram of recombinant virus construction. The *p48*KO bacmid was constructed by substituting *p48* in the AcBac-Syn (AcBac) bacmid with a bleoR cassette. The recombinant bacmids were constructed by integrating S-tagged *p48* or S-tagged mutant *p48* into the *hr4a* locus. (**B**) Transfection–infection assay. Sf9 cells were transfected with each recombinant bacmid. At 96 h post-transfection (p.t.), supernatants were collected and used to infect healthy Sf9 cells. Fluorescence microscopy images were captured at 96 h p.t. and 96 h post-infection (p.i.). (**C**) P0 viral titers of C141S, C229S, C320S, and *p48*Rep determined by the endpoint dilution method. Statistical analysis was conducted using Student’s t-test to compare the titer between each virus and the repair control. ns, not significant. ** *p* < 0.001. (**D**) Growth curve analysis. C229S, C320S, and *p48*Rep were analyzed at an MOI of 5, while C141S was analyzed at an MOI of 0.02. Statistical analysis was conducted using Student’s t-test to compare the titer at 96 h p.i. between each virus and the repair control. ns, not significant.

To analyze the role of the conserved cysteines for P48 function, we constructed bacmids with a *p48* knockout (*p48*KO), repaired *p48* (*p48*Rep), and a series of *p48* point mutations. As shown in Figure 3A, the *p48*KO was created using AcBac-Syn [15] as the backbone (AcBac), by replacing nucleotides (nt) 268 to 977 of *p48* with a bleomycin resistance gene (bleoR) cassette. The *p48*Rep and site-mutated bacmids were generated by transposition at the *hr4a* locus using the Bac-to-Bac system. The point (replacing cysteine with serine) mutations included conserved cysteines in the zinc finger motif (single-point mutants C127S, C130S, C138S, and C141S, and double-point mutants C127/130S and C138/141S), as well as two other conserved cysteines unrelated to the motif (C229S and C320S).

After the bacmids were created and verified by PCR and sequencing, they were used in transfection and infection assays (Figure 3B). Cells transfected with *p48*KO exhibited limited fluorescence at 96 h post-transfection (p.t.). This is consistent with previous studies showing that *p48* is crucial for BV production [4]. In contrast, *p48*Rep showed robust BV production, indicated by numerous fluorescent cells at 96 h p.t. and 96 h post-infection (p.i.). All zinc finger motif-related cysteine mutants, except C141S, failed to produce infectious BVs, as they did not result in fluorescent cells during the infection assay. The C141S mutant could produce infectious BVs, but the number of fluorescent cells was significantly lower than that of *p48*Rep. These findings suggest that the zinc finger motif is crucial for P48’s role in BV production. Conversely, the conserved cysteines C229S and C320S, not part of the zinc finger motif, demonstrated similar outcomes to *p48*Rep.

### 3.4. Zinc Finger Motif-Related Cysteines Are Critical for Transport of Nucleocapsids out of the Nucleus, ODV Envelopment, and OB Morphogenesis

Previous studies showed that deletion of P48 resulted in the failure of nucleocapsid transport out of the nucleus and disrupted the envelopment of ODV during baculovirus infection [2]. To investigate the effect of *p48* mutations on nucleocapsid transport and the morphogenesis of ODVs and OBs, Sf9 cells were transfected with recombinant bacmids and analyzed by transmission electron microscopy (TEM) at 72 h p.t. The mutants included two single-site mutations (C127S and C141S) and a conserved site mutation unrelated to the zinc finger motif (C229S), along with controls (*p48*KO, *p48*Rep, and AcBac).

**Figure 4:**
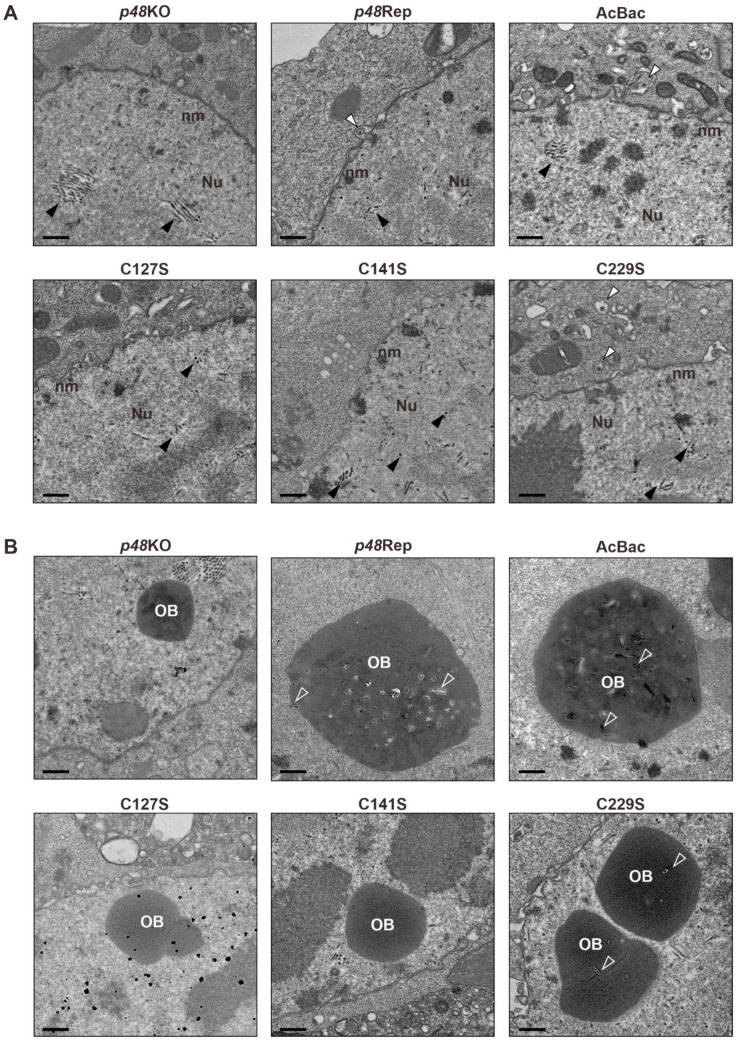
Transmission electron microscopy (TEM) analysis of zinc finger motif-related mutants and controls. (**A**) Representative TEM images showing the nucleocapsid transport events in each sample. Black arrowheads indicate nucleocapsids within the nucleus, while white arrowheads point to nucleocapsids being transported out of the nucleus. Scale bar, 500 nm. (**B**) Representative images showing the occlusion body (OB) morphogenesis of each sample. ODVs embedded in OBs are indicated by hollow arrows. Scale bar, 500 nm.

As shown in Figure 4A, no cytoplasmic BVs were observed in the *p48*KO samples, although nucleocapsids (black arrowheads) were present in the nuclei. In contrast, nucleocapsids being transported out of the nucleus (white arrowheads) were observed in the *p48*Rep and AcBac samples. Representative TEM images of single-site mutations (C127S and C141S) showed results similar to those of the *p48*KO samples, with no observable nucleocapsid transport out of the nucleus. However, the C229S mutation, which is unrelated to the zinc finger motif, displayed results similar to those of the *p48*Rep and AcBac samples, that is, the nucleocapsids being transported out of the nucleus. These results are consistent with the above findings that the cysteines of the putative zinc finger motif are crucial for BV production.

Additionally, as shown in Figure 4B, abnormal OBs were observed in the *p48*KO sample, which were empty and did not contain ODVs. Moreover, no enveloped ODVs were observed in the nuclei of the *p48*KO sample. In contrast, the normal OBs containing multi-nucleocapsid ODVs were observed in the *p48*Rep and AcBac samples. Empty OBs were also observed in the TEM images of single-site mutations (C127S and C141S), with no enveloped ODVs detected in the nuclei. OBs containing ODVs were observed in the C229S sample, but unlike the *p48*Rep and AcBac samples, they contained fewer ODVs and were predominantly single-nucleocapsid ODVs. These results indicate that the P48 zinc finger motif-related residues are crucial for ODV envelopment and OB morphogenesis. Additionally, C229 also plays an important role in ODV envelopment and OB morphogenesis.

## 4. Discussion

As a core gene, *p48* is essential for BV production and ODV envelopment, but its specific structure–function relationship remains unclear. In this study, the structure of P48 was predicted using AlphaFold, revealing a conserved putative zinc finger structure. Sequence alignment showed that these four sites are highly conserved in homologous proteins of P48 (Figure 1). Different from our sequence alignment results, a previous study by Wang et al. did not reveal the conservation of C127 and C130 [5]. The difference is likely due to the use of different sequence alignment algorithms: Wang et al. employed CLUSTAL X, while we used MAFFT. The function of the potential zinc finger motif was further investigated through mutation analysis. Our mutation analysis revealed that mutations in the key cysteines of the zinc finger motif impair P48’s functional roles in several critical steps in the viral life cycle, including nucleocapsid trafficking, BV production, ODV envelopment, and OB morphogenesis. Interestingly, among the four key cysteines involved in the Zn motif, the mutant C141S could produce infectious viral particles to some extent. This could be due to the presence of C143, which is located near C141 in AcMNPV and may substitute C141 to maintain the zinc finger motif function to a certain level. However, C141S showed a significantly reduced titer of BV, and consistently, we could hardly observe nucleocapsid trafficking events in the TEM results. These findings suggest that the zinc finger motif plays a pivotal role in the function of P48.

Zinc finger motifs are versatile structural domains that mediate interactions with DNA, RNA, and other proteins, playing important roles in transcriptional regulation, signal transduction, etc. [18]. P48 has been shown to interact with several host and viral proteins involved in nucleocapsid trafficking. For example, P48 interacts with Vps24 of the ESCRT-III complex [3] and the N-ethylmaleimide-sensitive factor of the SNARE machinery [19], which are host proteins involved in the nuclear egress of nucleocapsids. P48 also interacts with Ac76, Ac93, and Ac106, which are essential viral proteins for the nuclear egress of nucleocapsids and the formation of intranuclear microvesicles [20]. It would be interesting to investigate the role of the zinc finger motif of P48 in its interactions with these proteins.

Based on the number and type of amino acids involved in zinc coordination, zinc finger motifs are classified into different types, including C2H2, C4, and C3H1. The predicted structure model showed that P48 homologs encoded by alpha-, beta-, and gammabaculoviruses are structurally as conserved as the C4 type, whereas deltabaculovirus-P48 is a C3H1 type and is structurally different. The functional domain of P48 was predicted using the DALI and Foldseek servers based on their structural similarities with other identified enzymes. The results showed that P48 homologs encoded by alpha-, beta-, and gammabaculoviruses do not have structural similarity with known enzymes. However, CuniNPV-P48 contains a domain with structural similarity to the communication (COMM) domain of the tryptophan synthase β-subunit (TrpB1) [21]. This implies that the function of P48 may be related to its interaction with other proteins. However, the COMM-like domain is unique to CuniNPV-P48 and was not observed in the P48 homologs of other baculoviruses.

## 5. Conclusions

In summary, these findings demonstrate that the cysteines associated with a conserved zinc finger motif are critical for the functions of P48 in infectious BV production, nucleocapsid transport, ODV envelopment, and the embedding of ODV into OBs.

## Data Availability

The data presented in this study are available upon request from the corresponding author.
